# Design, synthesis and spectroscopic properties of crown ether-capped dibenzotetraaza[14]annulenes

**DOI:** 10.3762/bjoc.15.57

**Published:** 2019-03-11

**Authors:** Krzysztof Miroslaw Zwoliński, Julita Eilmes

**Affiliations:** 1 Faculty of Chemistry, Jagiellonian University, Gronostajowa 2, 30-387 Kraków, Poland

**Keywords:** crown ether, dibenzotetraaza[14]annulene, DBTAA, macrocycle, Schiff base

## Abstract

The first crown ether-capped dibenzotetraaza[14]annulenes (DBTAAs), featuring two macrocyclic binding sites fixed in a face-to-face orientation, were synthesized in satisfactory 26–28% isolated yields. Direct N-alkylation of 1,4,10-trioxa-7,13-diazacyclopentadecane by symmetric DBTAA derivatives bearing bromoalkoxy pendants proceed smoothly at a reasonable level of dilution (1.25 mM). The structures were fully characterized by HR-ESIMS, FTIR-ATR, ^1^H and ^13^C NMR spectroscopy and elemental analysis.

## Introduction

The design and synthesis of novel polycyclic receptor architecture is of fundamental importance, since model recognition studies contribute to much better understanding of complex biological systems [1]. Since the serendipitous discovery of crown ethers by Charles Pedersen in 1967 [2], there have been significant advances in the design and synthesis of sophisticated multidentate macrocycles with mixed-donor sites [3] and high selectivity towards the cationic organic species [4] and ions of certain metal elements [5]. Particulary, synthetic receptors featuring crown ether moieties often equipped with extra side arms bearing pendant functional groups [6–7], have attracted a great deal of interest due to their outstanding binding ability and offer promising applications in catalysis, bioinorganic, biomimetic and analytic chemistry [8]. Polycyclic architecture that incorporate crown ether moieties feature, confined space capable of displaying fascinating properties, different from those observed in bulk solution [9]. Therefore, the design and synthesis of functionalized, cage-like architecture containing converging binding sites arranged along the concave inner surface is in high demand [10]. Nevertheless, synthetic access to such a sophisticated polycyclic architecture is somewhat challenging and often requires the use of special techniques and apparatuses [11]. Particularly, the crown ether-capped (or ‘crown-capped’) porphyrins are synthesized conventionally under high-dilution conditions starting from two porphyrin building blocks bearing complementary functionalities, such as amine and either acyl chloride [12] or halomethyl [13], respectively.

**Figure 1:**
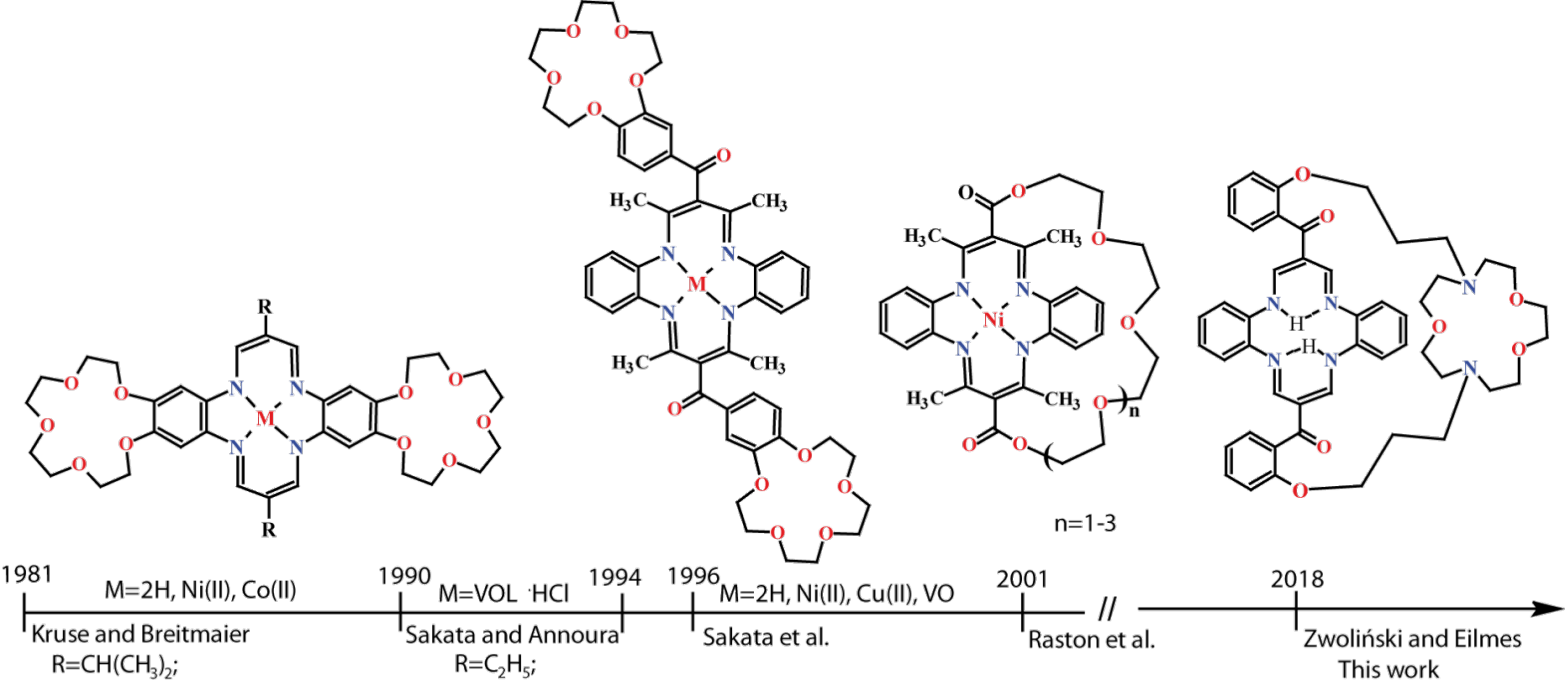
Timeline for the structure evolution of crown-capped DBTAAs (created on the basis of references [24–28]).

The family of dibenzotetraaza[14]annulenes (abbreviated hereafter as DBTAAs) belong to a class of quadridentate Schiff base macrocycles that display exceptional stability toward light, oxygen and water [14]. Thanks to basic similarities in their structure and physicochemical properties, DBTAAs emerged as non-pyrrolic surrogates of porphyrins, and have become a versatile platform for supramolecular [15], biomimetic [16–17], biological [18–19] and material chemistry [20–21]. Although much effort has been directed towards the study of crown ether-capped porphyrins [22] and sapphyrines [23], little research has been undertaken regarding DBTAAs functionalized with crown ether moieties (see Figure 1).

Kruse and Breitmaier [24] reported annulated DBTAA‘s containing benzo-15-crown-5 scaffolds fused with benzenoid rings of the macrocyclic core. Both nickel(II) and cobalt(II) complexes were prepared and their binding properties toward sodium and potassium ions were studied in addition to transition metals. Sakata et al. reported the syntheses and characterization of nickel(II), copper(II) [25] and oxovanadium(IV) [26] complexes of crown ether-annulated DBTAAs, which caused dimerization in the presence of alkali metal and ammonium ions. Sakata et al. reported the synthesis of peripherally functionalized DBTAAs with the two benzo-15-crown-5 scaffolds appended to the meso-benzoyl substituents [27]. Both crown ether-capped macrocycles in a form of free base and nickel(II) and cobalt(II) complexes showed cation and solvent-induced dimerisation. More recently, Raston et al. reported the synthesis of Goedken’s macrocycle tmtaa nickel(II) complex featuring a single oligo(ethylene glycol) chain strapped across the face of the DBTAA macrocyclic core [28]. However, to the best of our knowledge, there are no reports on ‘crown-capped’ dibenzotetraaza[14]annulenes.

## Results and Discussion

### Design principle and synthesis

To create polycyclic cage architecture, featuring two converging binding sites, capable of simultaneous recognition and cooperative binding of a variety of charged entities [29] or even whole ion pairs within the same superstructure [30], we decided to combine the two macrocyclic building blocks within the same rigidified architecture. Therefore, novel ‘crown-capped’ receptors **3a** and **3b** were designed to be composed of diaza-crown ether and DBTAA macrocycles. Arrangement of the two macrocyclic components in a face-to-face orientation create a central cavity lined by the two converging binding sites. We decided to make use of the previously reported DBTAA derivatives, bearing 3-bromopropoxy- [31] and 4-bromobutoxybenzoyl [32] pendants, since the presence of rigidifying benzoyl substituents protect the central cavity from collapsing. Simultaneously, the use of short but flexible aliphatic chains as part of the bridges make such receptors flexible enough to adjust the size of the crown cavity to the dimension of the charged entity. The new receptors **3a** and **3b** contain two apparently differentiated Lewis-basic binding sites, however, from a broader perspective, the nature of the DBTAA binding site can be easily switched into a Lewis-acidic one after appropriate metal insertion (e.g., with zinc(II) ions) [33].

**Scheme 1:**
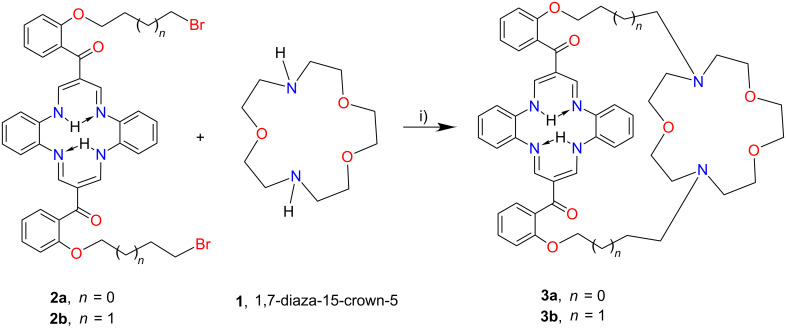
Synthesis of the crown-capped DBTAA receptors **3a** and **3b**. Reagents and conditions: i) Et_3_N, CH_3_CN, reflux, 24 h.

The crown-capped DBTAA’s were synthesized by refluxing 1,4,10-trioxa-7,13-diazacyclopentadecane (1,7-diaza-15-crown-5, kryptofix 21) with the previously reported bromoalkoxy derivatives **2a** and **2b** in anhydrous acetonitrile containing an excess of triethylamine for 24 hours (Scheme 1). The desired crown ether-capped receptors **3a** and **3b** were isolated following column chromatography as viscous oils in satisfactory 26–28% isolated yields. The use of either mixtures of acetonitrile and toluene (v:v, varied 4:1 to 1:4) as less polar reaction media did not improve the overall yields despite extension of the reaction time to 48 hours.

### Spectral characterization

The new compounds **3a** and **3b** have been fully characterized by FTIR-ATR, ^1^H and ^13^C NMR spectroscopy, high-resolution electrospray ionization mass spectrometry (HR-ESIMS) and elemental analysis, and the results are in agreement with expected structures (vide infra).

**Figure 2:**
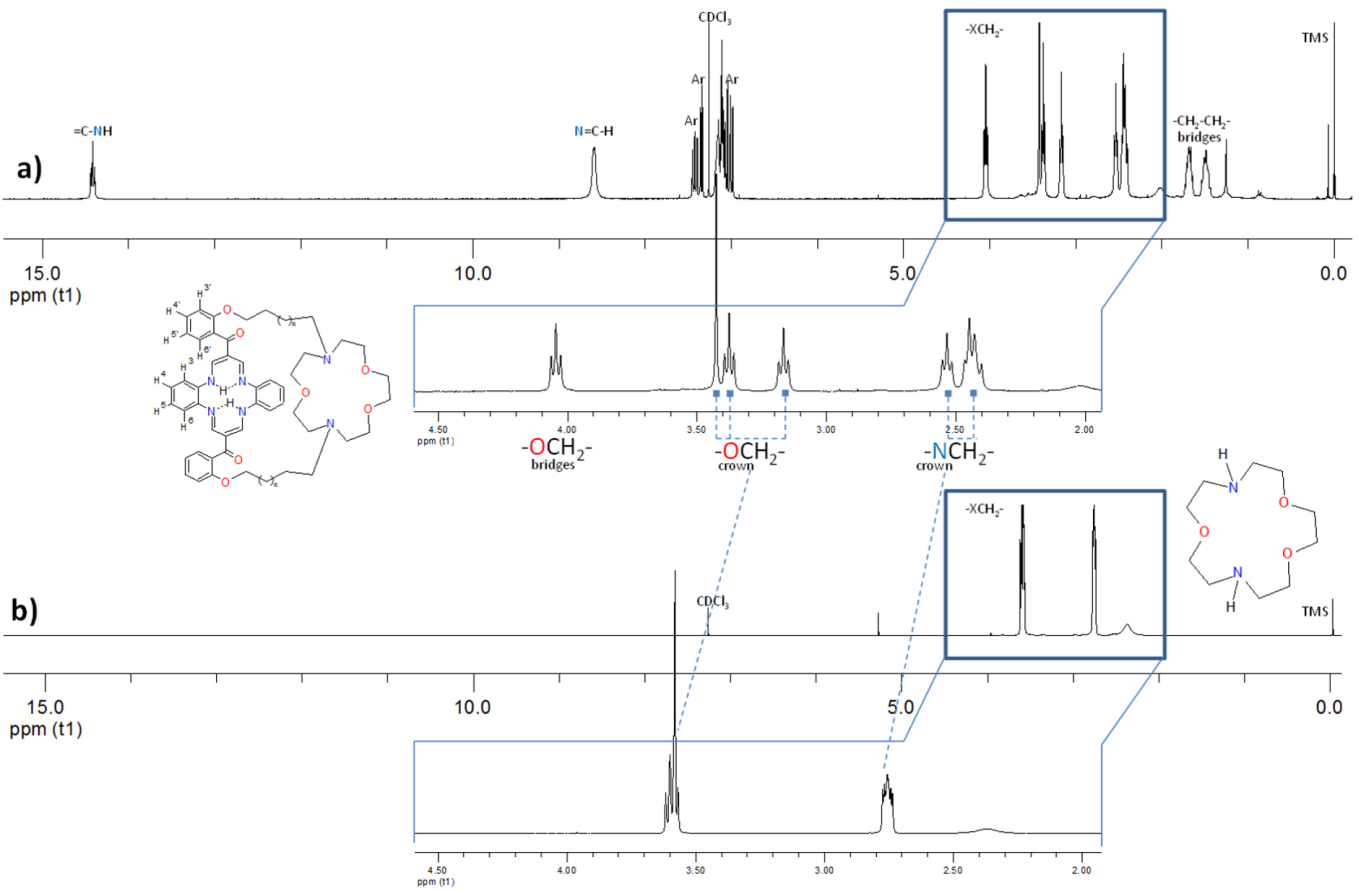
^1^H NMR spectra (298 K, CDCl_3_) of: a) crown ether-capped receptor **3b** and b) it’s corresponding parent 1,7-diaza-15-crown-5. The insets show an enlarged portion of ^1^H NMR spectra showing signals attributed to the crown ether ethylene protons.

Both the crown ether-capped compounds **3a** and **3b** are highly symmetrical as was evidenced by both the ^1^H and ^13^C NMR spectra. The ^13^C NMR spectra of **3a** and **3b** display only one characteristic resonance signal assignable to the carbonyl moieties at δ_C_ ≈193 ppm. Simultaneously, macrocyclic inner NH protons gave a well-resolved triplet at δ_H_ ≈14.4 ppm arising from the coupling with the olefinic H–C=N protons (*J* ≈6.7 Hz), observed as doublet at δ_H_ ≈8.6 ppm. Nevertheless, the most intriguing feature of the ^1^H NMR spectra of the crown ether-capped compounds **3a** and **3b** is the exceptionally large separation of proton resonance signals observed for the crown straps of **3b** (Figure 2a). ^1^H NMR spectra of both the *N*,*N'*-dimethyl [34] and *N*,*N'*-dibenzyl [35] derivatives of 1,7-diaza-15-crown-5, consist of two multiplets at δ_H_ 3.5–3.6 and 2.6–2.9 ppm, corresponding with oxo- and azaethylene proton resonances. Surprisingly, crown ether ethylene proton resonances are pronouncedly shifted downfield for the receptor **3b** as compared with the parent 1,7-diaza-15-crown-5 and the receptor **3a**. Although exact origin of such a difference needs to be further evaluated, it seems reasonable that the contraction of the bridging chains induces conformational changes that trigger constraints into the benzoyl ring rotation. The fingerprint of the oxoethylene proton resonances is composed of two triplets at δ_H_ 3.38 and 3.17 ppm and intense singlet at 3.43 ppm. At the same time, the fingerprint of the azaethylene proton resonances is composed of two triplets at δ_H_ 2.53 and 2.45 ppm; the latter is partially overlapped with proton resonances of aliphatic bridges. Receptor **3b** displays the properties of chemical shift reagents, similar to the previously reported crown ether-capped porphyrin [36] but of different origin.

Since, the macrocyclic dibenzotetraaza[14]annulene core is antiaromatic, and shows no diamagnetic ring current characteristic for porphyrins, the remarkable chemical shift differentiation observed for the crown strap shall be rationalized by its close spatial proximity to anisotropy cones spanning from aromatic substituent’s.

Moreover, vicinal coupling constants ^3^*J*_H-H_ found for the crown ethylene bridges fall within the range of 5.4–5.5 Hz, expected for gauche stereochemistry. Such a relaxed crown conformation is suitable for accommodation of large alkali and alkaline-earth cations.

The general features of FTIR-ATR spectra of receptors **3a** and **3b** are substantially similar to each other and almost undistinguishable from that of the corresponding parent α,ω-dibromoalkoxy-derivatives **2a** and **2b**. Slightly higher intensities are observed for absorption bands attributed to both the C–H (2924–2857 cm^−1^) and C–O–C (1246–1044 cm^−1^) stretching vibrations. The presence of strong absorption bands at ca. 1590 and 1560 cm^−1^, assignable to the olefinic and imine skeletal stretching modes, implies the macrocyclic scaffold integrity. The FTIR-ATR spectra consist also with the characteristic, but modest in intensity, the absorption band at ca. 1646 cm^−1^ attributable to the carbonyl moiety stretching vibrations.

The HR-ESIMS (positive ion mode) spectra of crown ether-capped DBTAA‘s **3a** and **3b** revealed base peaks of the pseudomolecular ions [M + H]^+^ at *m*/*z* 855.4467 and 827.4135, respectively, that are in a very good agreement with the theoretical isotopic distribution patterns.

## Conclusion

Creation of polycyclic architectures predisposed to perform higher forms of molecular behaviour is essential for advancing the art of biomimetic chemistry. Therefore, the first crown ether-capped DBTAAs, featuring a face-to-face arrangement of the macrocyclic components, have been synthesized in satisfactory 26–28% isolated yields and fully characterized based on HR-ESIMS, FTIR-ATR, ^1^H and ^13^C NMR spectroscopy and elemental analysis. These novel receptors are expected to bind both the transition, alkali and alkaline-earth metals, and also, after DBTAA subunit metalation the whole ion pairs. The binding properties of the crown ether-capped DBTAA receptors are under investigation and will be reported elsewhere.

## Experimental

### General procedure for the synthesis of crown ether-capped DBTAA’s **3a** and **3b**

1,7-Diaza-15-crown-5 (0.1 mmol, 22 mg) together with triethylamine (0.05 mL) was added to a suspension of appropriate α,ω-dibromoalkoxy macrocyclic derivative **2a** or **2b** (0.1 mmol) in 80 mL of anhydrous acetonitrile and stirred under reflux for 24 h. In due course, the orange precipitate gradually disappeared and a clear red solution was obtained. Afterwards, the reaction mixture was evaporated to dryness under reduced pressure and the crude residue was taken up in 20 mL of chloroform and washed with distilled water (2 × 50 mL). The organic phase was dried over anhydrous MgSO_4_, filtered through a glass filter, concentrated to a minimal volume and applied directly onto the top of a silicagel column equilibrated with chloroform. The column was firstly eluted isocratically with a mixture of chloroform and acetone (1:1, v/v) to remove any unreacted traces of α,ω-dibromoalkoxy macrocyclic derivatives **2a** or **2b**. The second band containing undesired site products was eluted isocratically with a mixture of chloroform and methanol (1:1, v/v). The third, main band, was eluted isocratically with a mixture of CHCl_3_/MeOH/NH_3aq_ (10:10:1, v/v/v), evaporated to dryness under reduced pressure, and kept in a vacuum overnight at ambient temperature to remove all volatiles.

**Crown ether-capped DBTAA macrocycle 3a: [7,16-{2,2'-[7,13-bis(3-propyloxy)-1,4,10-trioxa-7,13-diazacyclopentadecane]dibenzoyl}-5,14-dihydrodibenzo[*b*,*i*][1,4,8,11]tetraazacyclotetradecine]:** Orange oil. Isolated yield 26.1% (26 mg). ^1^H NMR (300 MHz, CDCl_3_, TMS, 298 K) δ 1.49 (m, 4H, CH_2_), 1.68 (m, 4H, CH_2_), 2.44 (m, 8H, NCH_2_), 2.53 (t, ^3^*J* = 5.4 Hz, 4H, NCH_2_), 3.17 (t, ^3^*J* = 5.5 Hz, 4H, OCH_2_), 3.38 (t, ^3^*J* = 5.4 Hz, 4H, OCH_2_), 3.43 (s, 4H, OCH_2_), 4.05 (t, *^3^J* = 5.6 Hz, 4H, CH_2_), 6.98–7.18 (m, 12H, H-3’,5’,3,4,5,6), 7.35 (dd, ^4^*J* = 1.7 Hz, ^3^*J* = 7.5 Hz, 2H, H-6’), 7.43 (ddd, ^4^*J* = 1.8 Hz, ^3^*J* = 7.4 Hz, ^3^*J* = 8.4 Hz, 2H, H-4’), 8.59 (d, ^3^*J* = 6.4 Hz, 4H, N=C-H), 14.44 (t, ^3^*J* = 6.6 Hz, 2H, NH); ^13^C{^1^H} NMR (75 MHz, CDCl_3_, TMS, 298 K) δ 23.68, 27.31, 53.35, 54.89, 56.25, 68.57, 70.03, 70.38, 110.44, 111.92, 115.58, 121.11, 126.49, 129.29, 129.45, 131.18, 137.23, 153.10, 155.55, 192.97 (C=O); FTIR (ATR) ν: 3069 (C=C), 2925 (C-H), 2857 (C-H), 1646 (C=O), 1589 (C=N), 1560 (C=C), 1488, 1446, 1286, 1246 (COC), 1137, 1102, 1044 (COC), 908, 822, 745, 660 cm^−1^; HR-ESIMS (+νe mode) *m*/*z* (%): [M + H]^+^ calcd for C_50_H_59_N_6_O_7_, 855.4445; found, 855.4467 (100), Δm = 2.2 ppm; anal. calcd for C_50_H_58_N_6_O_7_·0.5CHCl_3_: C, 66.31; H, 6.45; N, 9.19; found: C, 66.16; H, 6.58; N, 9.02.

**Crown ether-capped DBTAA macrocycle 3b: [7,16-{2,2'-[7,13-bis(4-butyloxy)-1,4,10-trioxa-7,13-diazacyclopentadecane]dibenzoyl}-5,14-dihydrodibenzo[*b*,*i*][1,4,8,11]tetraazacyclotetradecine]:** Red oil. Isolated yield 28.7% (16 mg). ^1^H NMR (300 MHz, CDCl_3_, TMS, 298 K) δ 1.82 (m, 4H, CH_2_), 2.53 (m, 12H, NCH_2_), 3.25 (t, ^3^*J* = 5.3 Hz, 4H, OCH_2_), 3.46 (s, 4H, OCH_2_), 3.47 (t, *^3^J* = 4.8 Hz, 4H, OCH_2_), 4.03 (t, ^3^*J* = 5.3 Hz, 4H, CH_2_), 6.97–7.18 (m, 12H, H-3’,5’,3,4,5,6), 7.41–7.47 (m, 4H, H-4’,6’), 8.59 (d, *^3^J* = 6.7 Hz, 4H, N=C-H), 14.44 (t, ^3^*J* = 6.7 Hz, 2H, NH); ^13^C{^1^H} NMR (75 MHz, CDCl_3_, TMS, 298 K) δ 26.90, 52.85, 53.87, 54.42, 66.51, 68.43, 70.09, 70.29, 110.56, 111.75, 115.07, 121.33, 126.39, 129.44, 129.83, 131.44, 136.83, 152.52, 155.40, 192.79 (C=O); FTIR (ATR) ν: 3069 (C=C), 2924 (C-H), 2862 (C-H), 1647 (C=O), 1589 (C=N), 1562 (C=C), 1489, 1449, 1287, 1247 (COC), 1139, 1101, 1045 (COC), 908, 823, 742 cm^−1^; HR-ESIMS (+νe mode) *m*/*z* (%): [M + H]^+^ calcd for C_48_H_55_N_6_O_7_, 827.4132; found, 827.4135 (100), Δm = 0.3 ppm; anal. calcd for C_48_H_54_N_6_O_7_^.^CH_2_Cl_2_: C, 64.54; H, 6.19; N, 9.22; found: C, 64.81; H, 6.32; N, 8.85.

## Supporting Information

File 1 Detailed descriptions of experimental methods and copies of original FTIR-ATR, HR-ESIMS, ^1^H and ^13^C NMR spectra for all new compounds.
