# Supplementary material for: Design, synthesis and spectroscopic properties of crown ether-capped dibenzotetraaza[14]annulenes
Source: Beilstein J Org Chem. 2019 Mar 11;15:617–22. doi: 10.3762/bjoc.15.57 (PMC6423591; doi:10.3762/bjoc.15.57)
Supplement: File 1 — Detailed descriptions of experimental methods and copies of original FTIR-ATR, HR-ESIMS, 1H and 13C NMR spectra for all new compounds. [file Beilstein_J_Org_Chem-15-617-s001.pdf]

BEILSTEIN JOURNAL OF ORGANIC CHEMISTRY

Supporting Information

for

Design, synthesis and spectroscopic properties of crown ether-capped dibenzotetraaza[14]annulenes

Krzysztof M. Zwoliński and Julita Eilmes

Beilstein J. Org. Chem. **2019,** *15,* 617–622. doi:10.3762/bjoc.15.57

Detailed descriptions of experimental methods and copies of original FTIR-ATR, HR-ESIMS, ^{1}H and ^{13}C NMR spectra for all new compounds

License and Terms: This is a supporting information file under the terms of the Creative Commons Attribution License (http://creativecommons.org/licenses/by/4.0). Please note that the reuse, redistribution and reproduction in particular requires that the authors and source are credited.
The license is subject to the *Beilstein Journal of Organic Chemistry* terms and conditions: (https://www.beilstein-journals.org/bjoc)

List of contents:

1. General information and experimental methods
2. Copies of FTIR-ATR, HR-ESIMS, ^{1}H and ^{13}C NMR spectra for new compounds (**3a** and **3b**)

1. General information and experimental methods:

All solvents and reagents were purchased from commercial sources (Sigma-Aldrich, Fluka, Lancaster) and used as received unless otherwise stated.

The NMR spectra were recorded using Bruker AMX (500 MHz) and Mercury Varian (300 MHz) spectrometers at 298 K in $CDCl_3$. The chemical shifts are reported in parts per million (ppm) and the coupling constants *J* are given in hertz (Hz). Data are reported as follows: chemical shift, multiplicity (s - singlet, br.s – broad singlet, d – doublet, m - multiplet), coupling constant and integration. ^{1}H NMR spectra were referenced to tetramethylsilane (TMS) as an internal reference standard (δ_H = 0 ppm). ^{13}C NMR spectra were referenced to the residual signal of $CDCl_3$ solvent (δ_C = 77.16 ppm).

High-resolution (HRMS) mass spectrometry experiments were performed on a Micromass LCT TOF MS (Time-Of-Flight) mass spectrometer using electrospray ionization technique and methanol as a spray solvent.

Fourier transform infrared FTIR ATR (Attenuated Total Reflectance) spectra were recorded at room temperature with a FTIR Thermo Fisher Nicolet IR200 spectrometer equipped with a diamond and operating in a single reflection mode.

Elemental analyses were conducted using Euro-EA (EuroVector) and VarioMicroCube microanalyzers. Samples were analyzed with standard parameters in a CHN mode.

2. Copies of FTIR-ATR, HR-ESIMS, ^{1}H and ^{13}C NMR spectra for new compounds (3a and 3b):

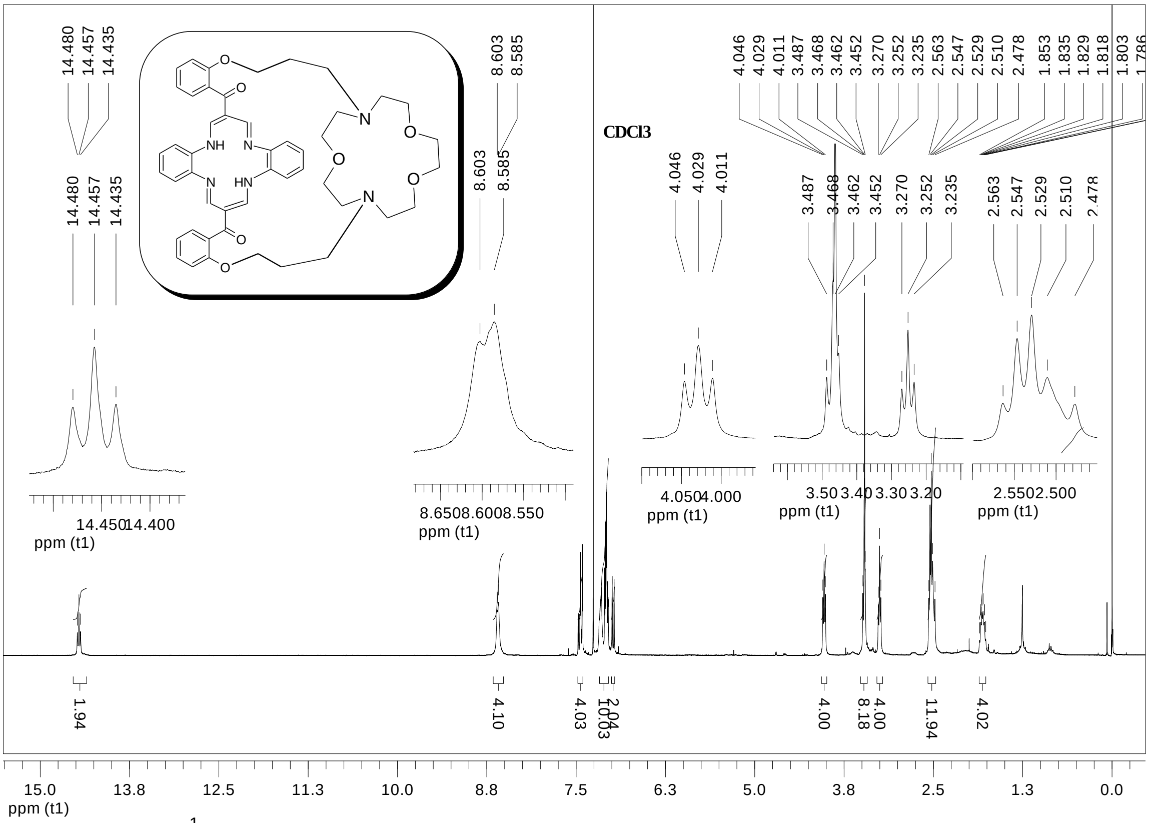

Figure S1: ^{1}H NMR spectrum of crown capped macrocycle **3a** (300 MHz, $CHCl_3$, 298 K).

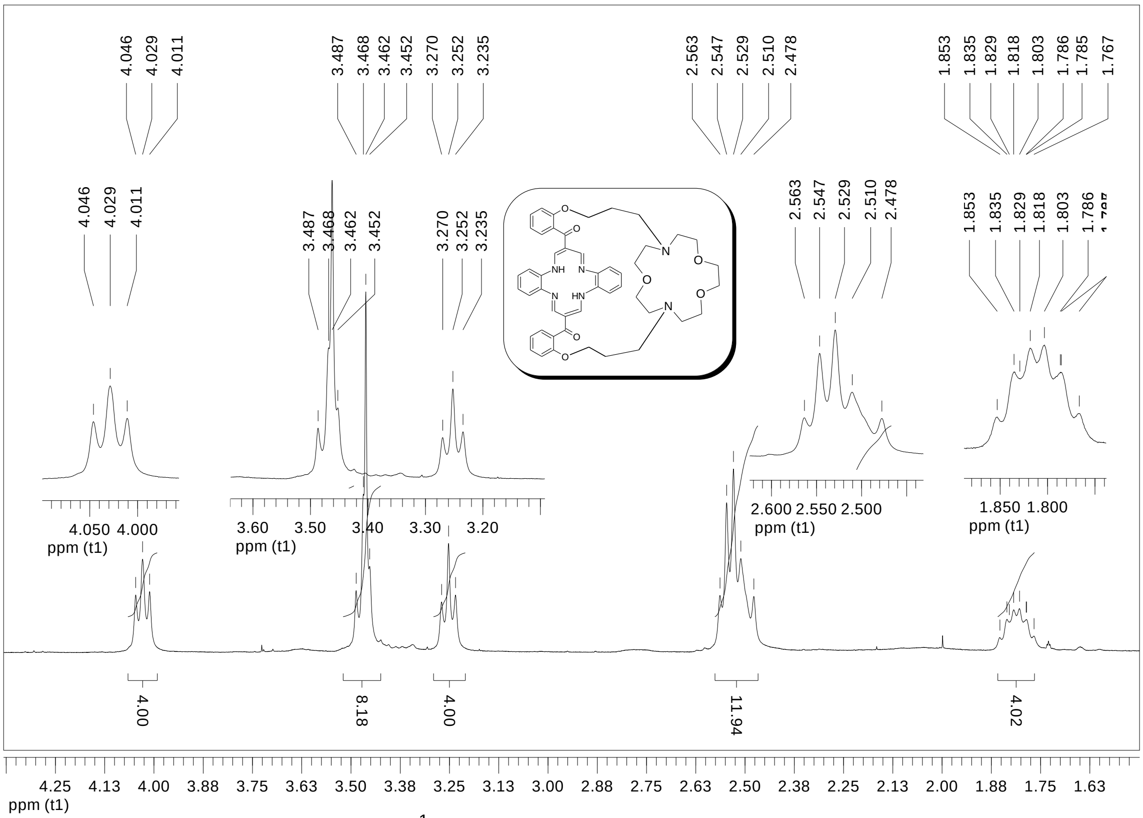

Figure S2: Expanded region of the ^{1}H NMR spectrum of crown capped macrocycle **3a** (300 MHz, $CHCl_3$, 298 K).

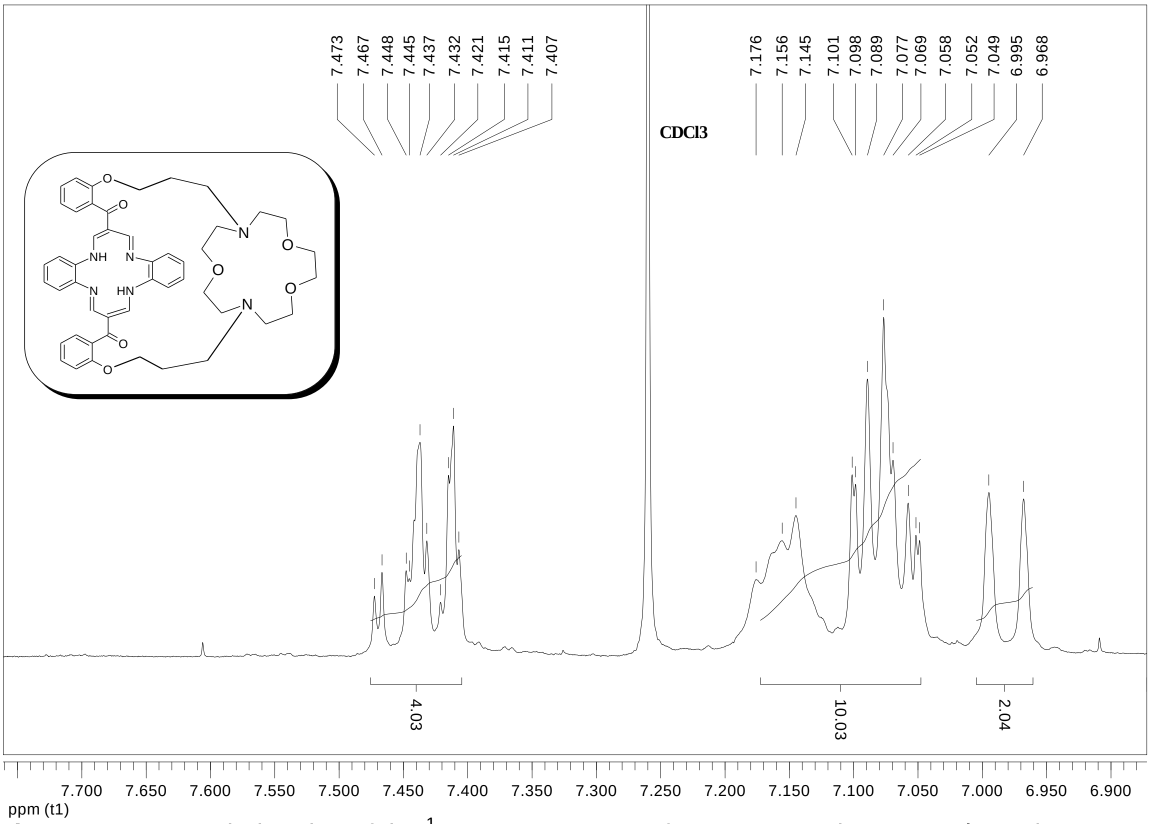

Figure S3:. Expanded region of the ^{1}H NMR spectrum of crown capped macrocycle **3a** (300 MHz, $CHCl_3$, 298 K).

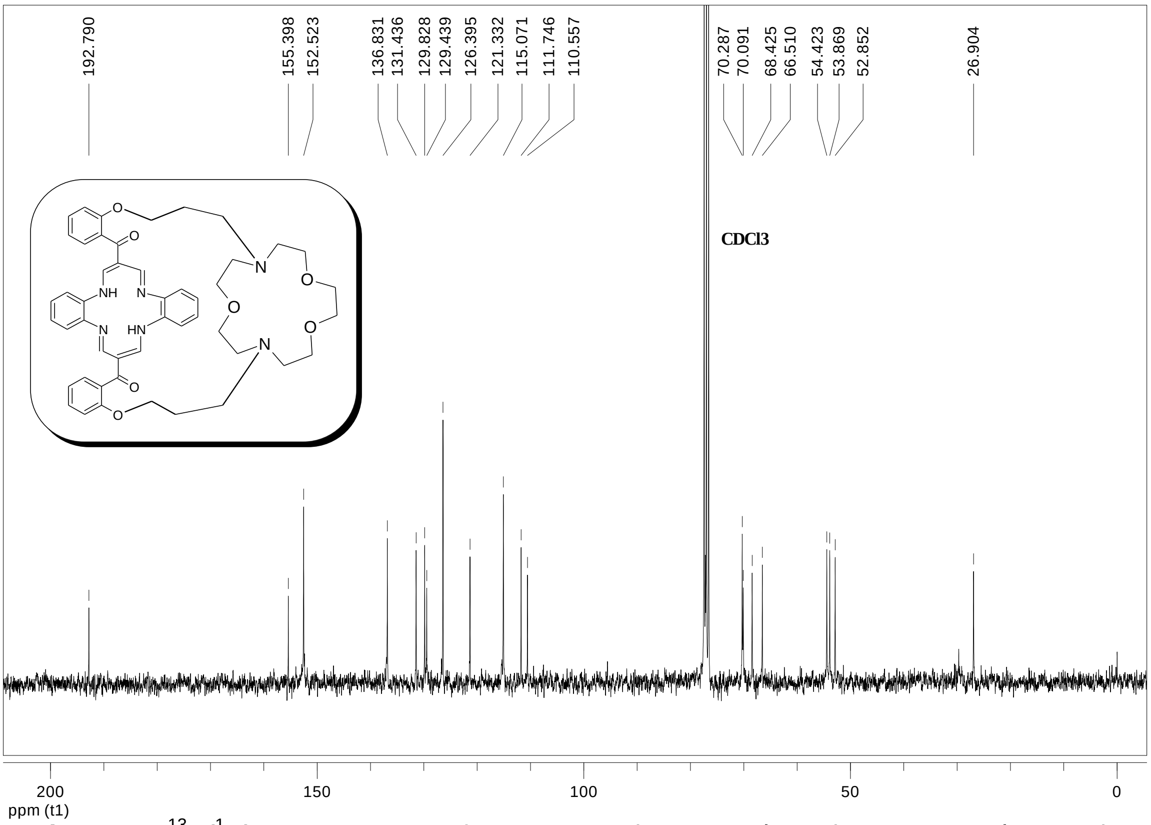

Figure S4: $^{13}C\{^1H\}$ NMR spectrum of crown capped macrocycle **3a** (75 MHz, $CHCl_3$, 298 K).

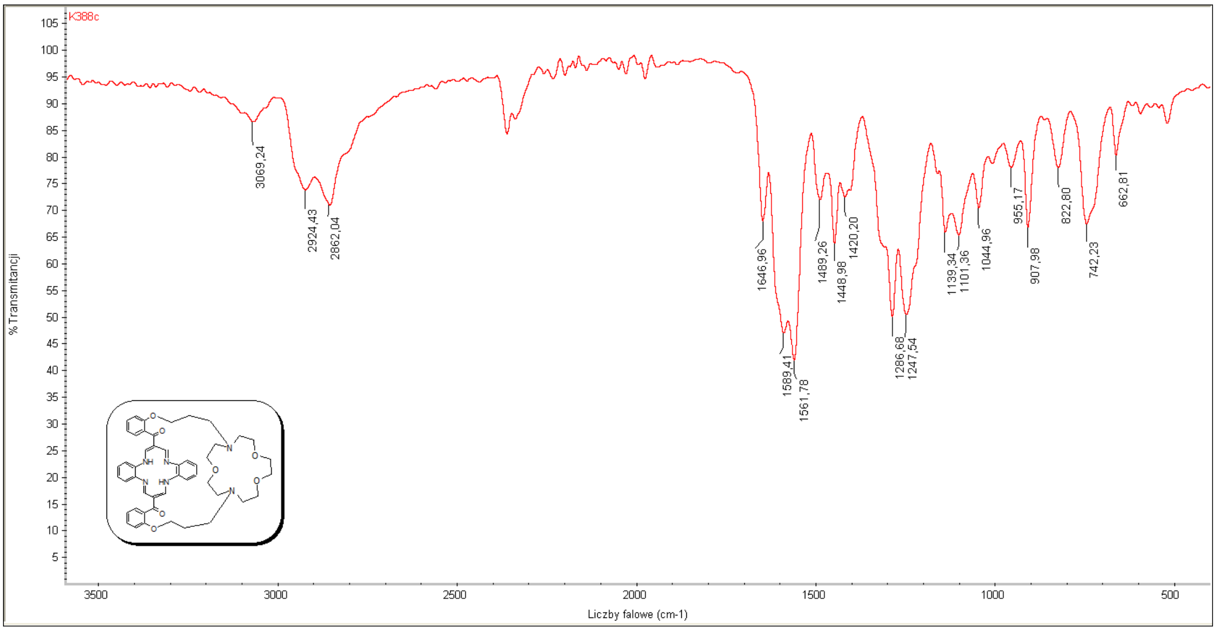

Figure S5: FTIR-ATR spectrum of crown capped macrocycle **3a** in a range of ν 400–3600 cm^{-1}.

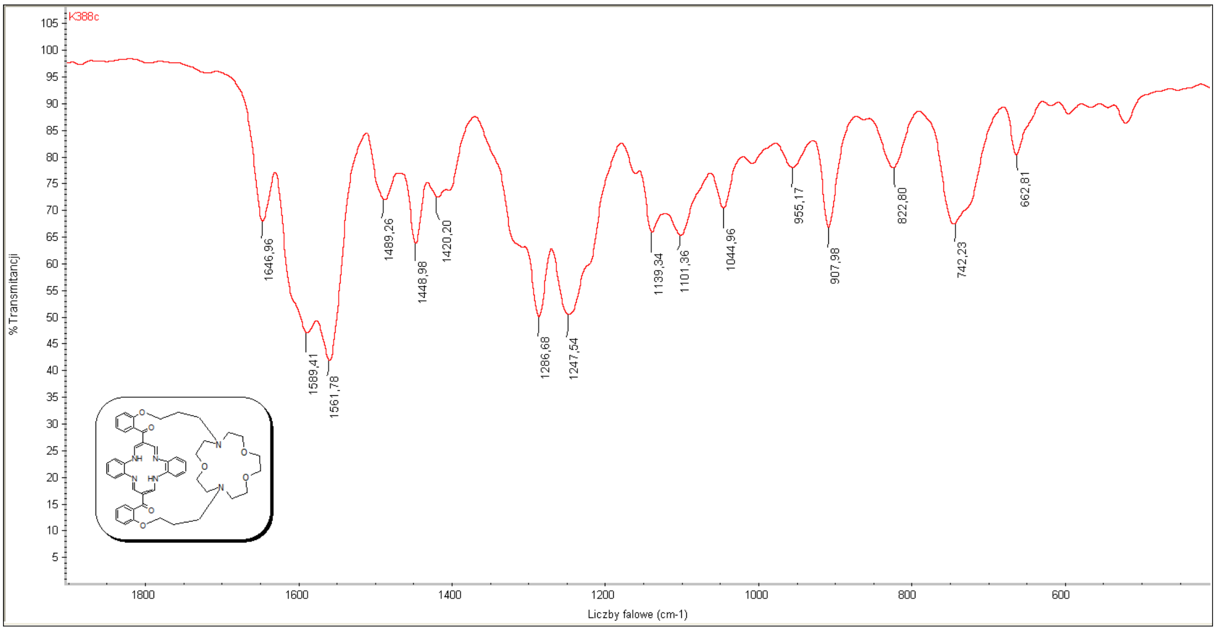

Figure S6: FTIR-ATR spectrum of crown capped macrocycle **3a** in a range of ν 400–1900 cm^{-1}.

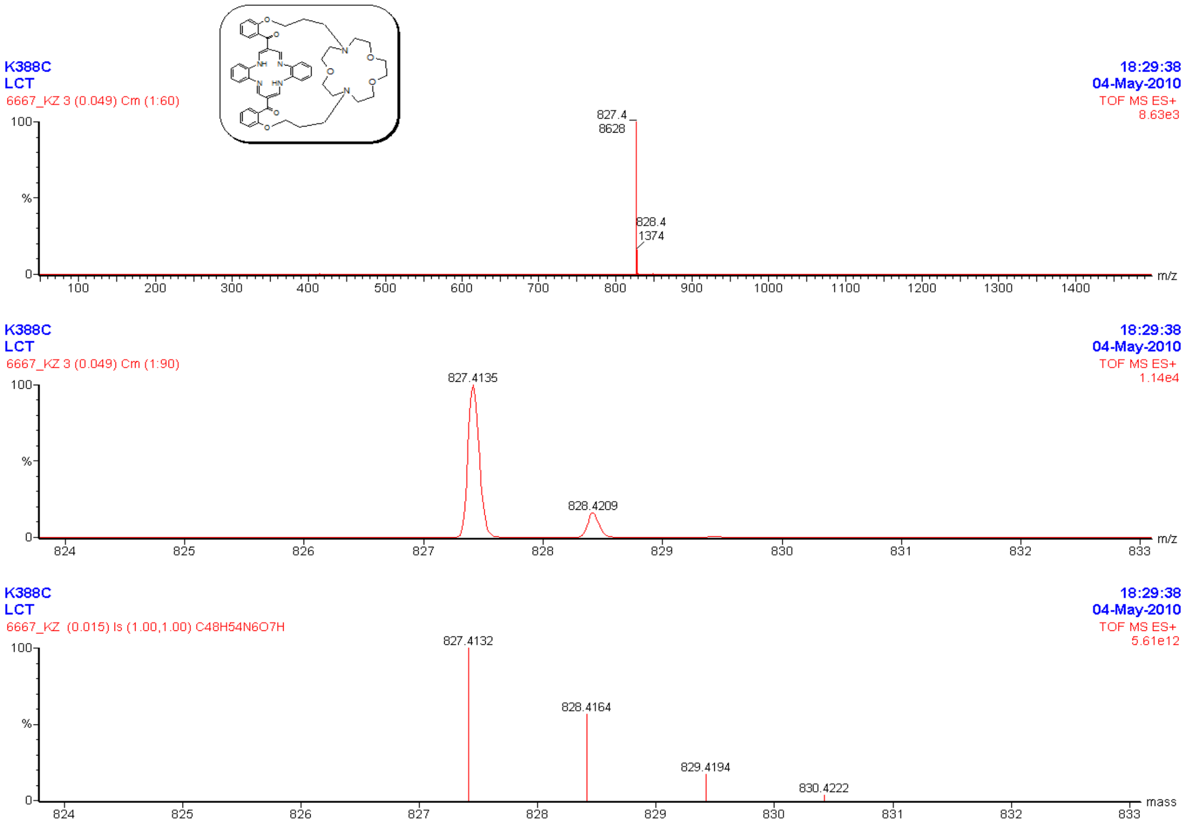

Figure S7: HR-ESIMS (positive mode) mass spectrographs of crown capped macrocycle **3a** showing the base peak of pseudomolecular ion $[M + H]^+$.

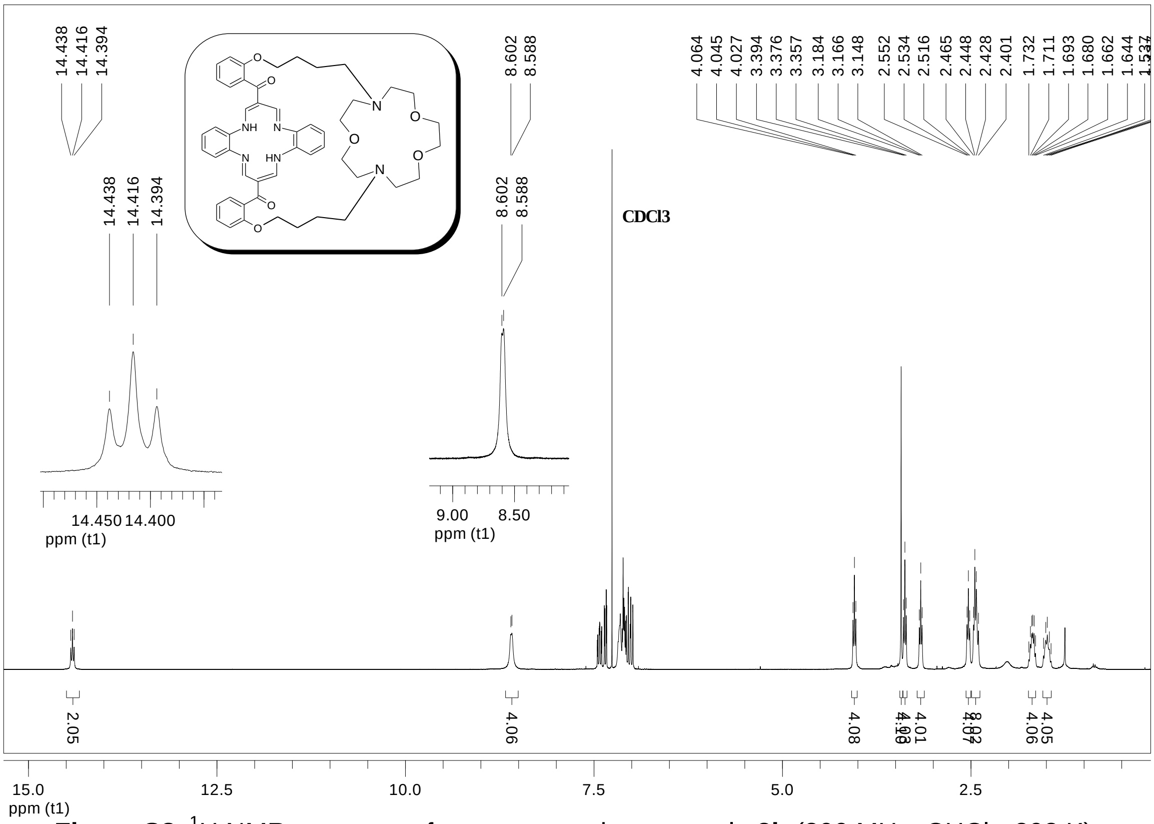

Figure S8: ^{1}H NMR spectrum of crown capped macrocycle **3b** (300 MHz, $CHCl_3$, 298 K).

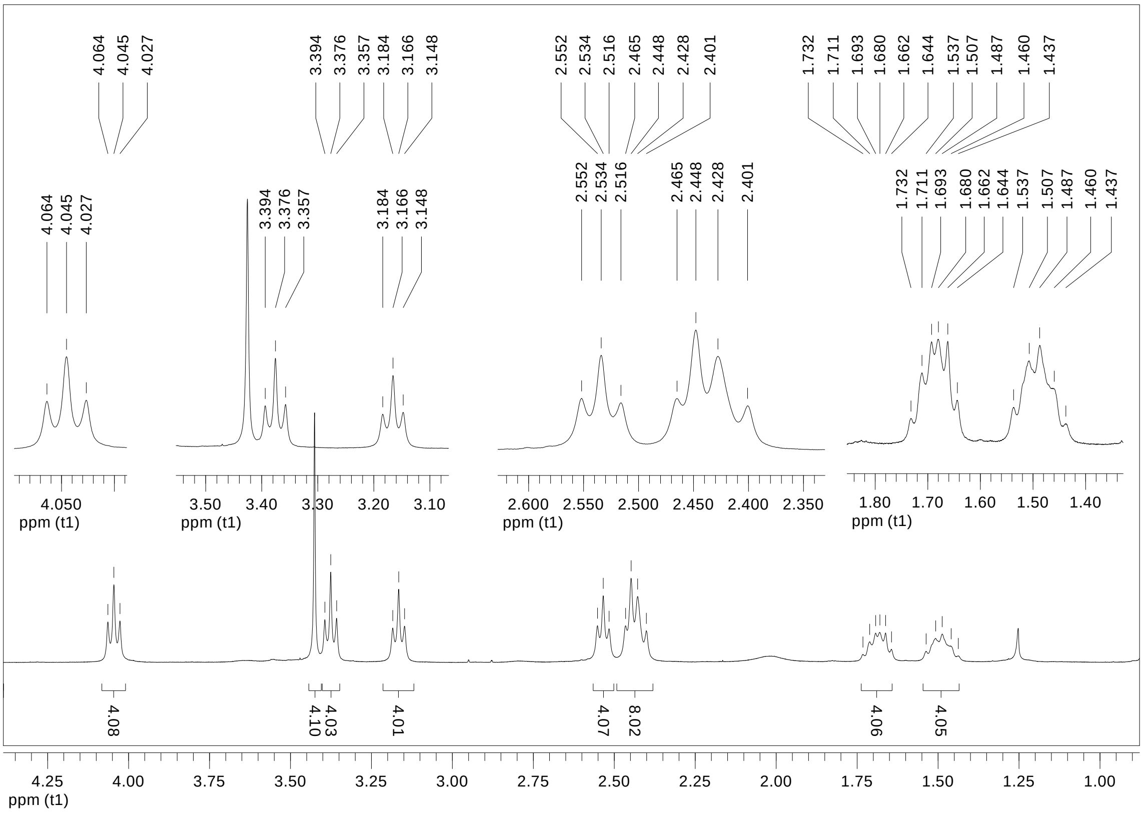

Figure S9: Expanded region of the ^{1}H NMR spectrum of crown capped macrocycle **3b** (300 MHz, $CHCl_3$, 298 K).

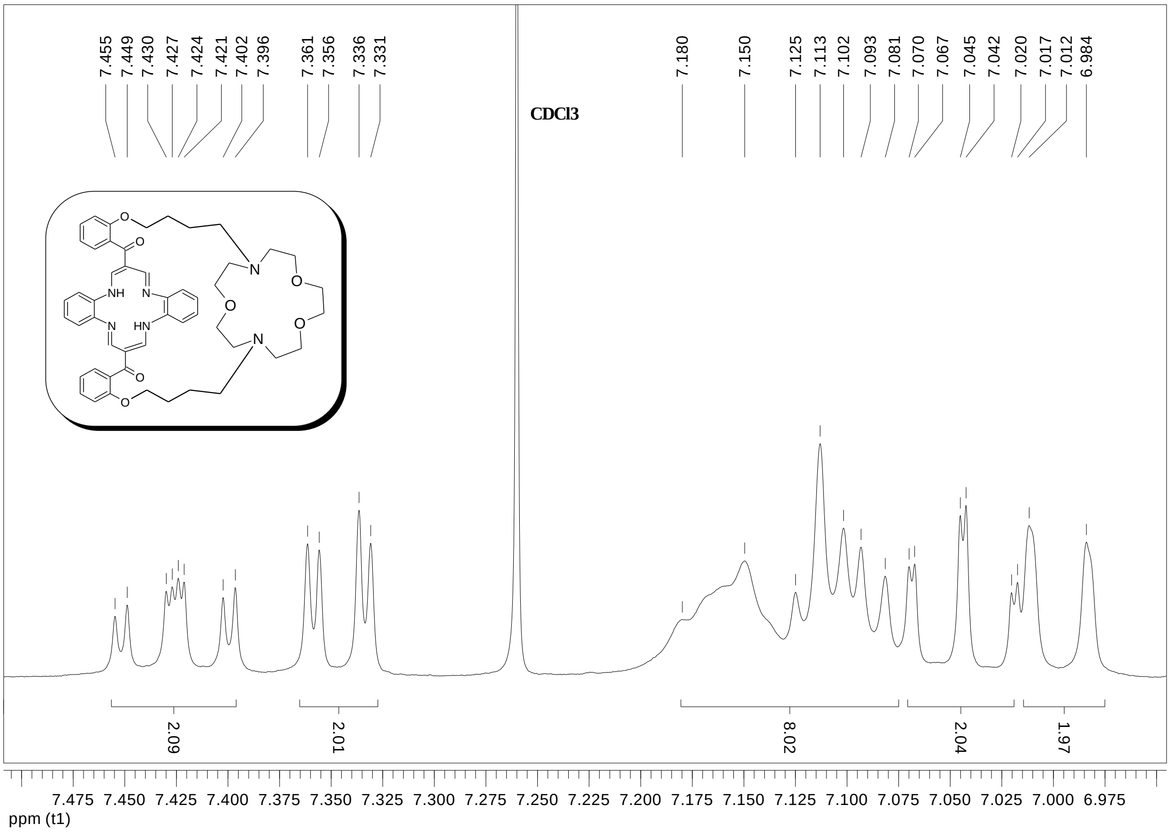

Figure S10: Expanded region of the ^{1}H NMR spectrum of crown capped macrocycle **3b** (300 MHz, $CHCl_3$, 298 K).

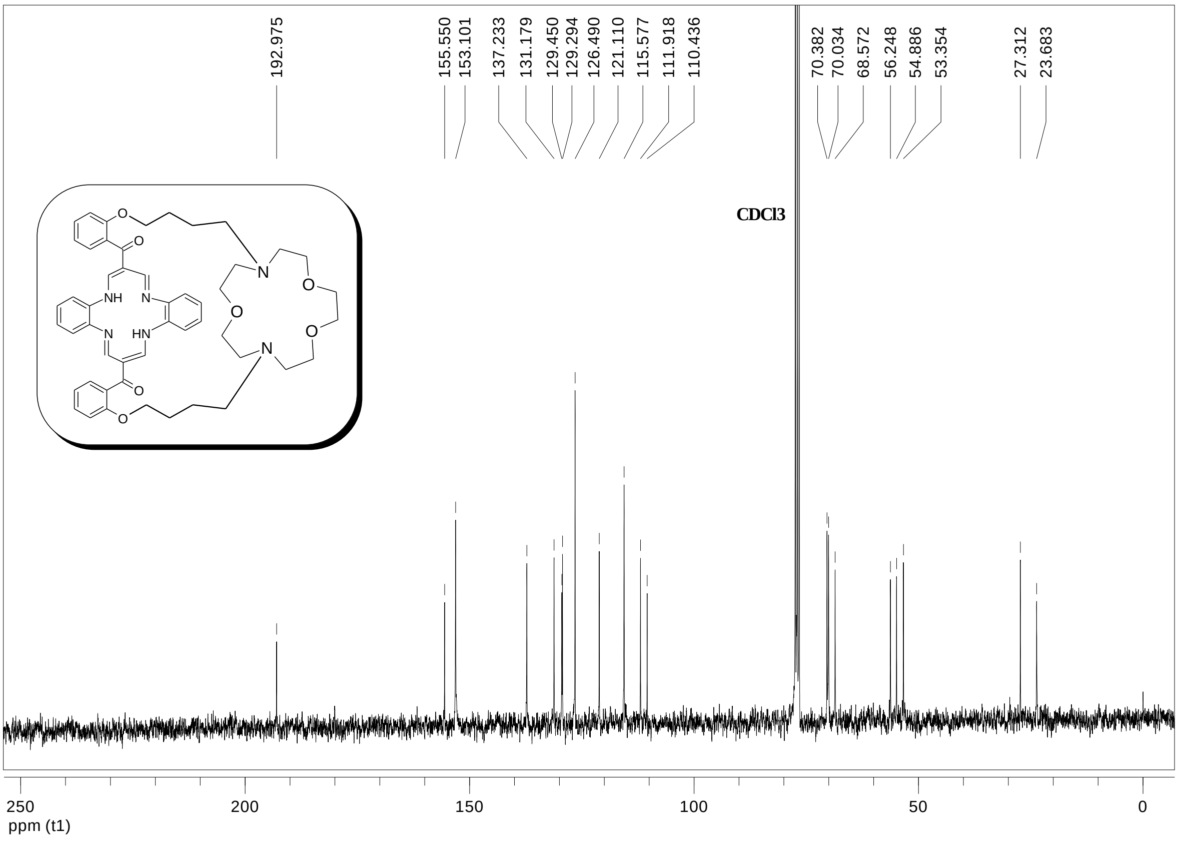

Figure S11: $^{13}C\{^{1}H\}$ NMR spectrum of crown capped macrocycle **3b** (75 MHz, $CHCl_3$, 298 K).

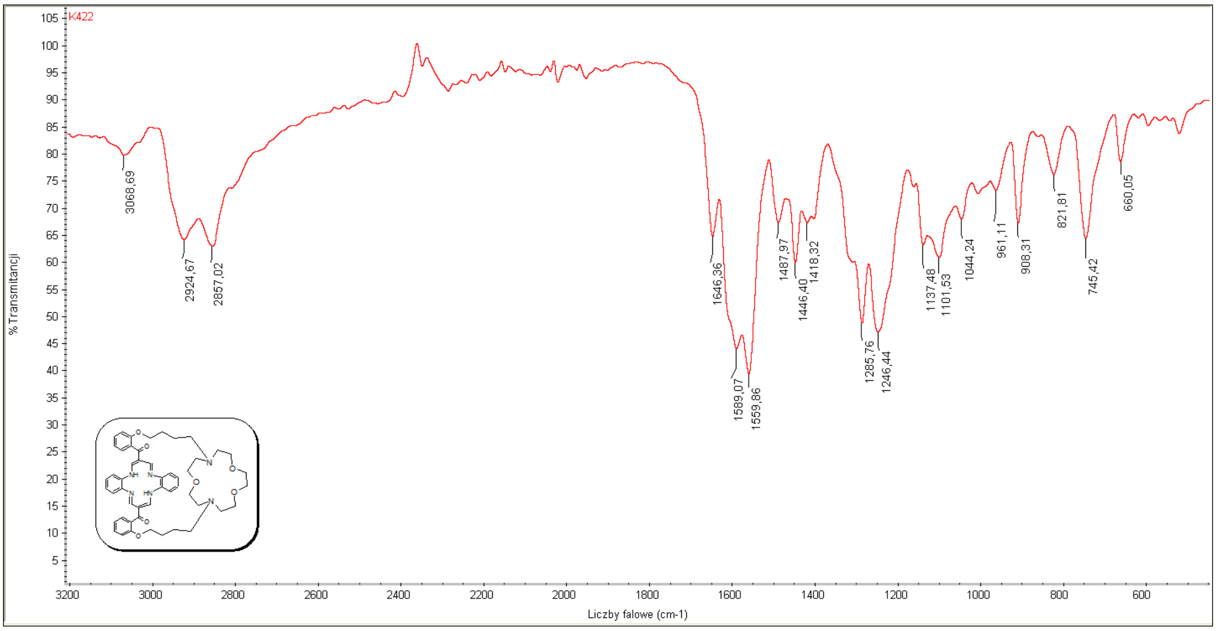

Figure S12: FTIR-ATR spectrum of crown capped macrocycle **3b** in a range of v 450–3200 cm^{-1}.

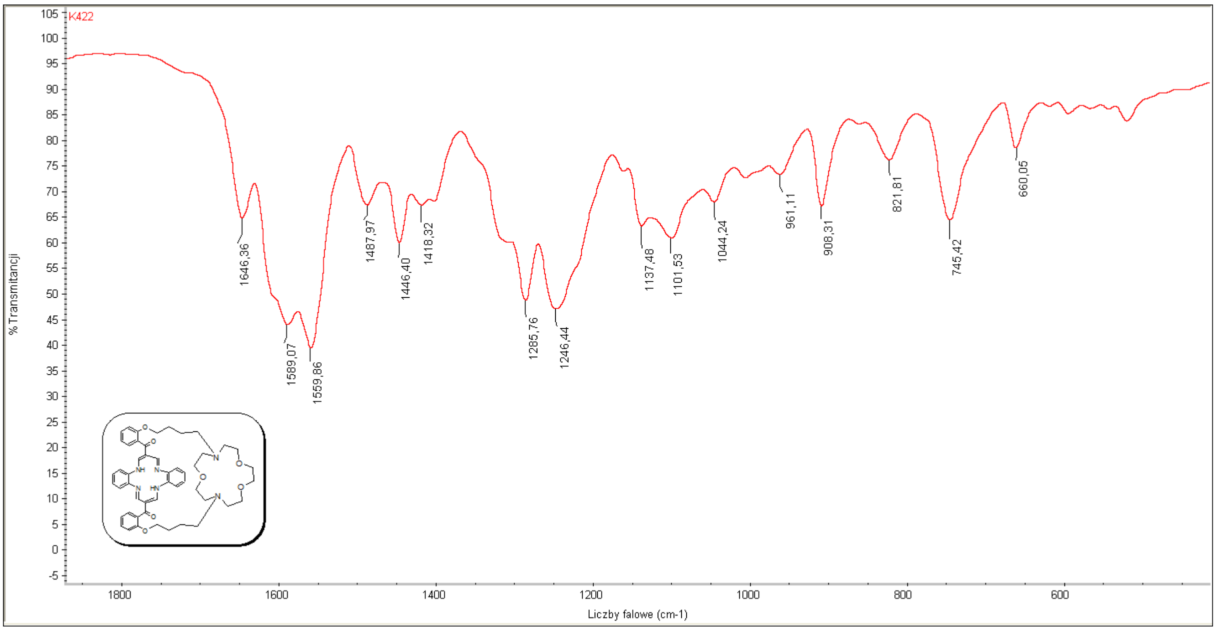

Figure S13: FTIR-ATR spectrum of crown capped macrocycle **3b** in a range of v 400–1900 cm^{-1}.

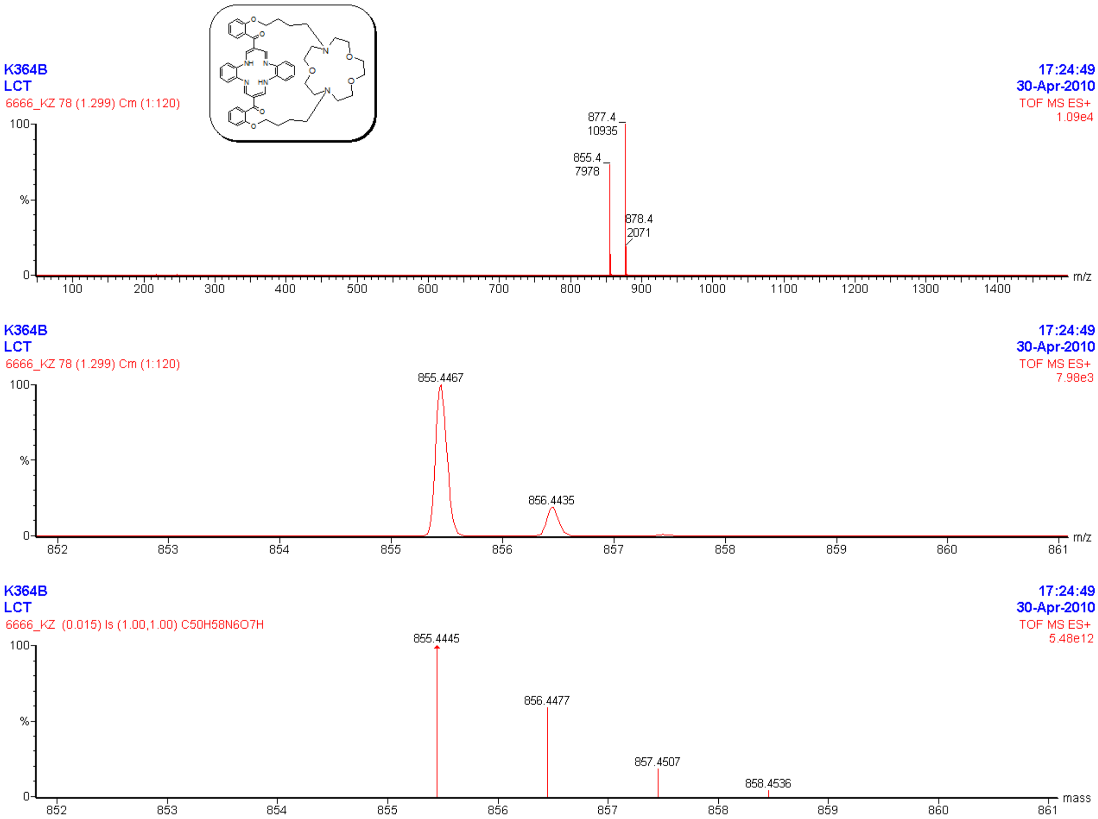

Figure S14: HR-ESIMS (positive mode) mass spectrographs of crown capped macrocycle **3b** showing the base peak of pseudomolecular ion $[M + H]^+$.
